# The IgG1/G2 subclass shift – a sensitive, tissue non-specific marker for malignancy. Diagnostic performance with squamous cell carcinoma of the head and neck

**DOI:** 10.1038/sj.bjc.6690283

**Published:** 1999-04

**Authors:** W Anderhuber, W Steinschifter, E Schauenstein, A Gotschuli, W Habermann, M Fischer, P Felsner, K Schauenstein

**Affiliations:** Department of Otolaryngology, Auenbruggerplatz 20, Graz, A-8010, Austria; Department of Biochemistry, , Halbärthgasse 5, Graz, A-8010, Austria; Department of General and Experimental Pathology, University of Graz, Heinrichstraβe 31, Graz, A-8010, Austria

**Keywords:** squamous cell carcinoma, head-neck region tumours, tumour marker, IgG subclasses

## Abstract

A significant decrease in %IgG1 accompanied by an increase in %IgG2 in total serum IgG has been previously reported as a highly sensitive marker for detecting early stages of carcinomas of various localizations. Here we investigated the question as to whether this phenomenon is also observed in sera of patients with squamous cell carcinoma of the head–neck region (SCC-HN), and to evaluate its diagnostic performance in the post-operative monitoring. Using quantitative affinity chromatography, serum concentrations of IgG1, IgG2 and total IgG were determined in 81 patients with different stages of primary and untreated SCC-HN, in 51 SCC-HN patients in post-therapeutical follow up, and in 33 patients with organ matched benign diseases. The data were compared with a total of 174 healthy controls. It was found that (i) 105 SCC-HN patients exhibited a mean value of 56.0 ± 0.7% IgG1, which likewise differed from healthy controls (63.2 ± 0.5) and benign diseases (61.5 ± 1.0) with *P* < 0.0005, (ii) sensitivities and specificities for discriminating primary malignancies from healthy controls were 70 and 74% respectively, and from benign diseases 65 and 76%, (iii) highest sensitivities and specificities were observed with post-therapeutic cases suffering from tumour recurrence (88% and 75%) or patients with distant metastases (87% and 86%), (iv) apparently tumour-free post-therapeutic patients showed a mean %IgG1 not different from the normal value. The decrease in %IgG1 accompanied by increased %IgG2 is an efficient, sensitive and early marker of SCC-HN, which appears particularly useful for the post-therapeutic monitoring. © 1999 Cancer Research Campaign


British Journal of Cancer (1999) 79(11/12), 1777–1781
© 1999 Cancer Research Campaign
Article no. bjoc.1998.0283

### The IgG1/G2 subclass shift – a sensitive, tissue non-specific marker for malignancy. Diagnostic performance with squamous cell carcinoma of the head and neck

**W Anderhuber[1], W Steinschifter[2], E Schauenstein[2], A Gotschuli[1], W Habermann[1], M Fischer[2], P Felsner[3] and K Schauenstein[3]**

[1]Department of Otolaryngology, Auenbruggerplatz 20, A-8010 Graz; [2]Department of Biochemistry, Halbärthgasse 5, A-8010 Graz; and [3]Department of General and Experimental Pathology, Heinrichstraβe 31, A-8010 Graz, University of Graz, Austria

**Summary** A significant decrease in %IgG1 accompanied by an increase in %IgG2 in total serum IgG has been previously reported as a highly sensitive marker for detecting early stages of carcinomas of various localizations. Here we investigated the question as to whether this phenomenon is also observed in sera of patients with squamous cell carcinoma of the head–neck region (SCC-HN), and to evaluate its diagnostic performance in the post-operative monitoring. Using quantitative affinity chromatography, serum concentrations of IgG1, IgG2 and total IgG were determined in 81 patients with different stages of primary and untreated SCC-HN, in 51 SCC-HN patients in post-therapeutical follow up, and in 33 patients with organ matched benign diseases. The data were compared with a total of 174 healthy controls. It was found that (i) 105 SCC-HN patients exhibited a mean value of 56.0 ± 0.7% IgG1, which likewise differed from healthy controls (63.2 ± 0.5) and benign diseases (61.5 ± 1.0) with $P < 0.0005$, (ii) sensitivities and specificities for discriminating primary malignancies from healthy controls were 70 and 74% respectively, and from benign diseases 65 and 76%, (iii) highest sensitivities and specificities were observed with post-therapeutic cases suffering from tumour recurrence (88% and 75%) or patients with distant metastases (87% and 86%), (iv) apparently tumour-free post-therapeutic patients showed a mean %IgG1 not different from the normal value. The decrease in %IgG1 accompanied by increased %IgG2 is an efficient, sensitive and early marker of SCC-HN, which appears particularly useful for the post-therapeutic monitoring.

**Keywords:** squamous cell carcinoma; head-neck region tumours; tumour marker; IgG subclasses

Despite high physiological variations in absolute serum concentrations, the distribution of IgG subclasses within total serum IgG in healthy persons is strictly adjusted to 60–70% IgG1, 20–30% IgG2, 5–8% IgG3, and 0.7–4% IgG4. In previous reports it was shown that patients with malignant diseases of various tissue origin exhibit a characteristic and highly significant alteration in the subclass composition of serum IgG, consisting in a reduction in %IgG1 and an increase in %IgG2. This phenomenon has been reported in patients suffering from carcinomas of the female breast (Kronberger et al, 1994), various locations of the female reproductive tract (Schauenstein et al, 1996), and of the colo-rectal region (Schauenstein et al, 1997), and it turned out to exhibit diagnostic sensitivities and specificities comparable to, or – particularly at early tumour stages – far higher than conventional serological tumour markers. Patients with benign tumorous or inflammatory diseases of the respective organs differed in their mean %IgG1 and %IgG2 values from cancer patients with the same high significance as did healthy controls.

Analogous results were obtained in experimental animal models. In rats it was found that the transplantation of the Yoshida hepatoma or Walker 256 carcinosarcoma cell lines led to an identical IgG subclass shift affecting IgG2b and IgG2a (Weblacher et al, 1993). The data obtained with this model revealed that this tumour-associated phenomenon essentially requires the presence of living, actively dividing tumour cells, that the shift becomes significant long before an exponential tumour growth, and that it is detectable also in the surface expression of the affected IgG subclasses on peripheral blood B lymphocytes (Maninger et al, 1994).

In this study we present for the first time results of %IgG1 and %G2 as obtained with patients suffering from squamous cell carcinomas of the head–neck region (SCC-HN). It should be noted that, with this type of malignancy, until now no efficient conventional serological marker has been available, particularly for the detection of post-therapeutic recurrences and/or metastases.

*Received 17 May 1998*
*Revised 23 September 1998*
*Accepted 9 October 1998*

Correspondence to: E Schauenstein

### MATERIALS AND METHODS

### Patients

A total of 81 patients were investigated after admission to the Department of Otolaryngology of the University Hospital Graz, consisting of 12 female (average age, 58) and 69 male patients (average age, 60), all suffering from primary, histopathologically verified SCC-HN. These patients were investigated as to %IgG1/IgG2 prior to any treatment, except for diagnostic biopsy. Table 1 shows the distribution of the patients according to characteristics of primary tumours (UICC TNM Classification of Malignant Tumors, 1992). Seven of the patients with advanced tumours were found afflicted with distant metastases.

**Table 1** Distribution of patients with primary tumours according to tumour burden

| | Tumour diameter | *n* |
|---|---|---|
| T1 tumour restricted to one area | ≤ 2 cm | 18 |
| T2 tumour in more than one area; in carcinomas of the larynx: no fixation of the vocal cord | 2–4 cm | 19 |
| T3 tumour diameter > 4 cm or fixation of vocal cord or hemilarynx in carcinomas of the larynx and hypopharynx | > 4 cm | 13 |
| T4 tumour exceeding organ borders, infiltrating neighbouring structures | | 31 |

A second group was investigated consisting of a total of 51 cancer patients who were in observation for 1–5 years after a combined treatment consisting of radical surgery, irradiation with an average dose of 60 Gy and a platin-based chemotherapy. These patients were monitored by local inspection and sonography every 3 months of the first 2 post-operative years, and every 6 months thereafter. Furthermore, they were examined once per year by computer tomography or magnetic resonance for local recurrence, as well as radiography of the thorax and sonography of the upper abdomen for distant metastases. Any suspicious findings were further investigated by biopsy and histological analysis. In 27 patients of this group no tumour could be detected (T0) at the time of investigation, whereas 22 showed local recurrent disease. Five patients of the latter group had developed distant metastases in addition to local recurrence, two of the patients had distant metastases only.

Finally, a total of 33 patients were included who were afflicted with diverse benign diseases of the head and neck, such as chronic infections of the paranasal sinuses, chronic tonsillitis, chronic laryngitis and also lateral and/or median cysts of the neck. The data of all these experimental groups were compared with the ones of 174 healthy controls (both sexes, age matched).

### Sampling

Blood (4–5 ml) taken from the forearm vein was allowed to clot for 15 min and centrifuged at 3000 rpm. Since subclass IgG1 had been shown earlier rapidly to form autoaggregates (Maninger et al, 1996) interfering with its detection, all determinations had to be performed not later than 60 min after sampling.

### Quantitation of subclasses IgG1, IgG2 and total IgG by affinity chromatography

The method was recently described (Schauenstein et al, 1997). In brief, 200 µl of serum was applied to a column (HiTrap, 1 ml, Pharmacia, Uppsala, Sweden) filled with Protein A Sepharose High Performance (Pharmacia, code-no. 17-0402-01); after removal of all non-binding components with phosphate–citrate buffer pH 7.0, IgG2 and IgG1 were sequentially eluted with the same buffer, but with pH-values of 4.7 and 4.3 respectively. Total IgG was eluted from a second serum aliquot (100 µl) with glycine–HCl buffer, pH 2.7, after passage through a 1 ml HiTrap

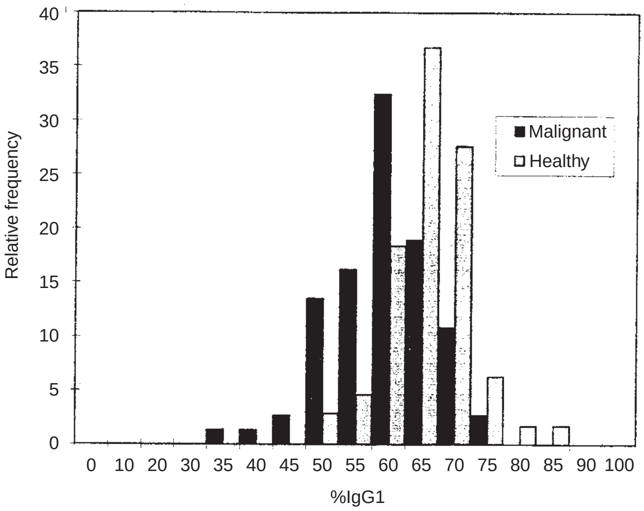

**Figure 1** Statistical distributions of single values of %IgG1, pretherapeutically determined in sera of 74 patients with SCC-HN (T1–T4), compared with 174 healthy controls column filled with Protein G Sepharose High Performance (Pharmacia, code-no. 17-0404-01). Protein concentrations in all eluates were determined at 280 nm, using a specific extinction coefficient for IgG, ε′ of 1.58 [1000 $g^{-1}$ $cm^2$] (Schauenstein et al, 1986).

### Statistical analysis

Single values obtained with all experimental groups and healthy controls showed normal distributions, as shown in Figure 1, for primary cancer patients vs. healthy controls. Accordingly, significances of the differences between mean values were calculated with the two-sided Student’s *t*-test, and cut-off values were calculated as the arithmetic means of mean values obtained from two groups to be compared. Diagnostic sensitivities and specificities were defined as percentages of patients with %IgG1 smaller, and of healthy controls with %IgG1 greater than the cut-off value respectively. ROC plots were calculated with the LABROC 1 program by Charles E Metz, Department of Radiology, The University of Chicago, 5841 South Maryland Avenue, Chicago, IL 60637–1470, USA.

### RESULTS

Table 2 summarizes the results obtained with patients carrying primary SCC-HN and with the cases in the post-therapeutic monitoring. The result is analogous to those previously reported for carcinomas of other localizations, i.e. a significant decrease in %IgG1 accompanied by an increase in %IgG2 within total IgG, as compared with benign diseases or healthy controls. Also in accordance with previous experiences, the tumour-associated changes in %IgG1 and %IgG2 were quantitatively not very high yet of highest significance, which is due to both the small biological variations of relative IgG1 and G2 mean values and the high accuracy of measurements (interassay variations of ±1–2%). No quantitative dependence on size and extent was observed with primary tumours, hence cases with T1 tumours exhibited the same

© Cancer Research Campaign 1999

**Table 2** %IgG1 and %IgG2 in total serum IgG, obtained with patients suffering from untreated primary SCC-HN, local recurrence or distant metastases respectively, cured tumour-free patients and cases of benign diseases of the HN region, in contrast to healthy controls

| Probands | *n* | mg IgG | ± s.e.m. | %IgG1 | ±s.e.m. | %IgG2 | ±s.e.m. |
|---|---|---|---|---|---|---|---|
| All cancer patients | 105 | 10 | 0.3 | 56[a] | 0.7 | 24.4[a] | 0.7 |
| Primary untreated SCC-HN (T1–T4) | 74 | 9.6 | 0.4 | 56.8[a] | 0.9 | 23.4[d] | 0.9 |
| Primary SCC-HN with distant metastases | 7 | 12.5 | 0.5 | 54.6[a] | 1.3 | 25.4[f] | 1.4 |
| Primary SCC-HN (T1) | 18 | 8.4 | 0.6 | 55.9[a] | 1.1 | 23.3[e] | 1.6 |
| Local recurrence | 16 | 10.4 | 1 | 54.9[a] | 1.4 | 26.8[d] | 2 |
| Local recurrence with distant metastases | 6 | 11 | 1.2 | 49.6[a] | 3.3 | 30.5[d] | 2.8 |
| Distant metastases without primary tumour or local recurrence | 2 | 13.9 | | 48.9 | | 31 | |
| All patients with distant metastases | 15 | 11.4 | 0.6 | 52.9[a] | 1.5 | 27.2[d] | 1.4 |
| Post-operative tumour-free (T0) | 27 | 8.4 | 0.5 | 61.9 | 0.9 | 23.9 | 1.5 |
| Benign diseases | 33 | 8.4 | 0.4 | 61.5[b,c] | 1 | 21[g,h] | 1.2 |
| Healthy controls | 174 | 10.3 | 0.2 | 63.2 | 0.5 | 20.4 | 0.5 |

[a]Different from healthy controls ($P < 0.0005$). [b]Different from malignant cases ($P < 0.0005$). [c]Different from primary untreated SCC-HN ($P = 0.0005$). [d]Different from healthy controls ($P = 0.0005$). [e]Different from healthy controls ($P = 0.05$). [f]Different from healthy controls ($P = 0.0025$). [g]Different from malignant cases ($P = 0.01$). [h]Different from primary untreated SCC-HN ($P = 0.05$).

significance as more advanced stages. In contrast, secondary disease consisting in local recurrence or distant metastasis showed a clear-cut increase in the effect, which is reflected in increased sensitivities and specificities (Table 3).

The histograph in Figure 1 shows the normal distributions of the single values of %IgG1 of healthy controls and primary tumour patients, which allows calculation of cut-off values, sensitivities and specificities as described in the Methods section. Identical distributions were seen with the other groups investigated (not shown). The sensitivity and specificity of %IgG1 for diagnosing SCC-HN are illustrated as ROC-plot in Figure 2. A clear-cut increase in the diagnostic performance is observed from primary tumours to local recurrences and metastatic disease. This quantitative dependence is also expressed with the second parameter, i.e. the increase in %IgG2, as shown in Figure 3. While no quantitative differences in the IgG1/G2 shift were noted between primary tumours of different stages (not shown), the decrease in %IgG1 and increase in %IgG2 became most obvious with local recurrences and distant metastases.

### DISCUSSION

SCC-HN predominantly affects males, whereby a variety of environmental irritants have been implicated, notably cigarette smoke (Maran et al, 1993). As head and neck cancers in general are easily accessible to inspection and tissue sampling, serological tumour markers are not of great relevance to the primary diagnosis in most of the cases (Dreyfuss et al, 1992). Thus, the primary need for a serological marker in head and neck cancer is certainly in the post-therapeutic monitoring, i.e. the early and sensitive diagnosis of local recurrence, and of distant metastases (Dreyfuss et al, 1992; Gleich et al, 1996), e.g. in lung, liver or bone, where tissue sampling is often not possible or dangerous. The criteria that should be met by a reliable and useful tumour marker are (Clasen et al, 1988): (i) a high sensitivity to early tumour stages, (ii) a high specificity for malignant growth, (iii) an easy and reproducible evaluation in daily routine practice, and (iv) the test should be rapid and inexpensive. Until now numerous tumour-derived substances have been evaluated as diagnostic indicators of tumour presence in untreated patients, but, with few exceptions, most of these direct markers proved to be only of limited clinical usefulness owing to extremely high biological variabilities, limited specificities for malignant proliferation, and low sensitivities for detecting early tumour stages. Particularly for SCC-HN no sufficiently sensitive non-invasive tumour marker was available until now (Fischbach et al, 1990). Squamous cell carcinoma antigen (SCC-A) was considered a promising candidate in aiding diagnosis and monitoring of patients with SCC-HN, but the diagnostic

**Table 3** Sensitivities and specificities obtained with %IgG1, discriminating SCC-HN from healthy controls or benign diseases

| | vs. Healthy controls | | vs. Benign diseases | |
|---|---|---|---|---|
| Probands | Sensitivity | Specificity | Sensitivity | Specificity |
| All cancer patients | 75 | 78 | 70 | 76 |
| Primary untreated SCC-HN | 70 | 74 | 65 | 76 |
| Local recurrence | 88 | 75 | 69 | 79 |
| Patients with distant metastases | 87 | 86 | 80 | 82 |

© Cancer Research Campaign 1999 

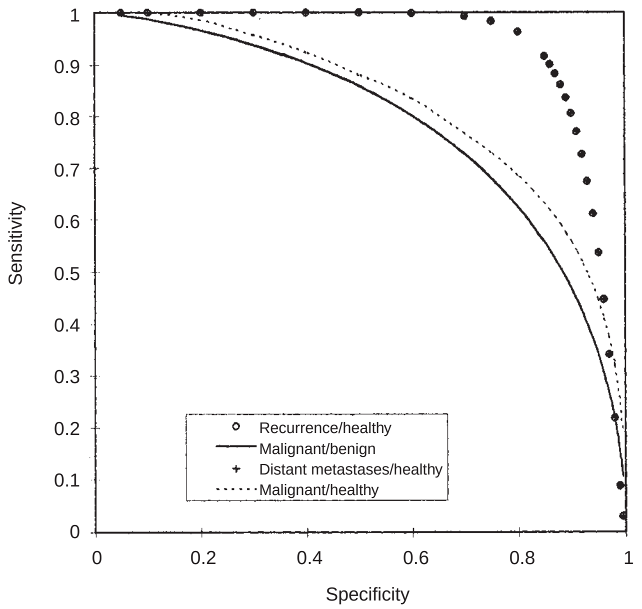

**Figure 2** ROC plots (sensitivities vs. specificities), obtained with %IgG1, comparing 105 malignant cases of SCC-HN with healthy controls and with 33 cases of benign diseases, and 22 cases with recurrent SCC-HN and 23 cases with distant metastases with healthy controls

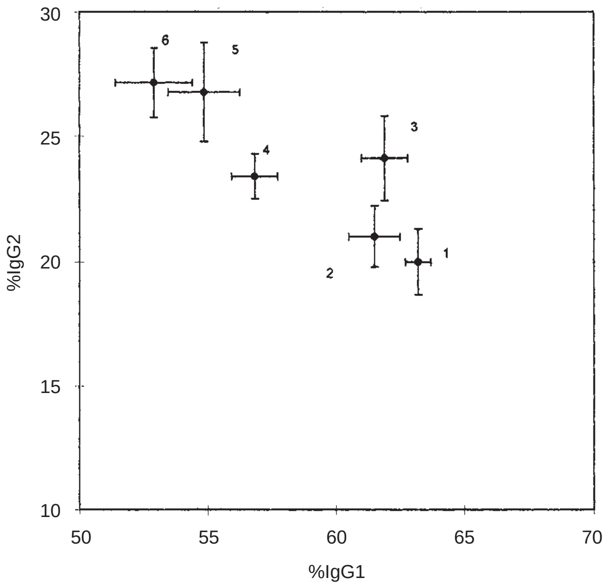

**Figure 3** Mean values of %IgG2 (ordinate) and of %IgG1 (abscissa) with the respective s.e.m., obtained with 174 healthy controls (1), 33 benign cases of the HN-region (2), 23 tumour free post-therapeutic patients T0 (3), 74 cases with primary, untreated SCC-HN, all stages (4), 16 patients with recurrent SCC-HN (5) and 15 patients with distant metastases (6)

performance finally turned out to be poor. SCC-A was described to be elevated (> 2 ng $ml^{-1}$) in 12–15% (Walther et al, 1990), 28% (Clasen et al, 1988), 33% (Dreyfuss et al, 1992), 38% (Fischbach et al, 1990; Koch et al, 1989) or 44% (Eibling et al, 1989) of SCC-HN patients. These results are in good agreement with our own unpublished experiences, where only 26% of a total of 322 SCC-HN patients were found to be positive. Other serological markers, such as platelet-derived growth factor (PDGF) or carcino-embryonic antigen (CEA) did fail as screening markers for primary SCC-HN. They may be useful, perhaps, in combination with other markers for monitoring purposes (Gleich et al, 1996; Silverman et al, 1976).

The present data suggest the changes in %IgG1 and %IgG2 as a useful serological tumour marker for detecting primary or recurrent and/or metastatic SCC-HN. It should be stressed once more that it is the specific alteration of the subclass pattern, independent from absolute quantitative changes of total serum IgG, that gives the relevant and highly sensitive information. Diagnostic sensitivities and specificities were so far calculated on the bases of the change in %IgG1 only. A bivariate analysis including %IgG2 may further enhance the discriminating efficiency.

Concerning the specificity of the IgG subclass shift for malignant proliferation, it is stated that a similar change in the relative concentrations of IgG1 and IgG2 has not been described with any other pathological condition. Patients afflicted with benign diseases (benign tumours or inflammations) of the tissues so far investigated sometimes showed a similar trend (Kronberger et al, 1994; Schauenstein et al, 1996; Schauenstein et al, 1997), but consistently differed from cancer patients with the same level of significance as did healthy controls. Even though our experience at present does not indicate any significant association of the IgG1/G2 shift with other common diseases, further studies are under way to examine this parameter in selected immunopathological conditions.

Whereas conventional serological markers directly correspond to tumorigenic products, the shift in %IgG1/G2 represents an indirect marker, consisting in a change of the host's immune system due to the presence of malignant tumours. Evidence for this notion has first been obtained in the rat model where, in parallel to the change in IgG subclass serum proteins, a similar shift was detected in the distribution of surface expressed IgG subclasses on B cells in the peripheral blood (Maninger et al, 1994). This suggests that the presence of a malignant process interferes with the regulation of the biosynthesis of IgG subclasses at the B-lymphocyte level. Very recent preliminary data obtained with a quantitative reverse transcriptase polymerase chain reaction to detect IgG1 and IgG2 specific mRNA suggest that the same may be true in humans. Peripheral blood lymphocytes of patients suffering from gynaecological malignancies were found to exhibit a drastically reduced ratio in the messages for IgG1 vs IgG2, as compared with healthy controls (Felsner et al, submitted for publication). Experiments are presently under way to examine the hypothesis that malignant tumours may modulate the IgG subclass biosynthesis by the expression of certain cytokines and/or their soluble receptors, which are involved in IgG subclass regulation (Kawano et al, 1994). Whatever the mechanisms are, the regulation of IgG subclasses apparently is extremely sensitive to respective signals derived from malignant cells, which is reflected by the high sensitivity of the phenomenon, particularly for early stages of various tumours in humans (Kronberger et al, 1994; Schauenstein et al, 1996, 1997) including SCC-HN (see present data), and in the rat model (Weblacher et al, 1993). An attractive feature of the IgG subclass shift as tumour marker are the extremely small variation ranges of the single values in normal controls and tumour patients,

 © Cancer Research Campaign 1999

as compared with several orders of magnitude often observed with conventional tumour markers. This fact, together with the normal distributions of the single values allows for simple statistical evaluations, without logarithmic transformation or non-parametric tests.

Until now the shift in %IgG1/G2 was reported as a phenomenon occurring with tumours of various tissue origin. In the framework of the present study, an apparent specificity for squamous cell carcinoma emerged, as a group of patients ($n$ = 13) suffering from head and neck tumours of other histopathological types, including large cell carcinoma, adenocarcinoma, poorly differentiated carcinoma, cylindrical cell carcinoma, and malignant lymphoma, exhibited mean values for %IgG1 of 64 ± 1.5 and for %IgG2 of 18.0 ± 1.5, which are both clearly in the normal range. Further studies are needed to confirm this finding, and to delineate further the specificities of the phenomenon for different tumours derived from other tissues.

### ACKNOWLEDGEMENTS

This study was supported by a grant from the Austrian Ministry of Science and Research.

### REFERENCES

Clasen B, Roettger D, Senekowitsch R and Menz E (1988) Squamous cell carcinoma antigen (SCC) – Derzeitige klinische Wertigkeit eines Tumormarkers bei Kopf-Hals-Karzinomen, Zwischenergebnisse einer prospektiven Studie nach 12 Monaten. *Laryng Rhinol Otol* **67**: 420–425

Dreyfuss AI, Clark JR and Andersen JW (1992) Lipid-associated sialic acid, squamous cell carcinoma antigen, carcinoembryonic antigen and lactic dehydrogenase level as tumor markers in squamous cell carcinoma of head and neck. *Cancer* **70**: 2499–2503

Eibling CDE, Johnson JT and Wagner RL (1989) SCC-RIA in the diagnosis of squamous cell carcinoma of the head and neck. *Laryngoscope* **99**: 117–124

Fischbach W, Meyer Th and Barthel K (1990) Squamous cell carcinoma antigen in the diagnosis and treatment follow-up of oral and facial squamous cell carcinoma. *Cancer* **65**: 1321–1324

Gleich LL, Srivastava L and Gluckmann JL (1996) Plasma platelet-derived growth factor: preliminary study of a potential marker in head and neck cancer. *Ann Otol Rhinol Laryngol* **105**: 710–712

Kawano Y, Noma T and Yata J (1994) Regulation of human IgG subclass production by cytokines. IFN-γ and IL-6 act antagonistically in the induction of human IgG1 but additively in the induction of IgG2. *J Immunol* **153**: 4948–4958

Koch T, Eiffert H and Spindler MB (1989) Die Relevanz des neuen Tumormarkers SCC (Squamous Cell Carcinoma Antigen) für die Diagnostik und Verlaufskontrolle von Plattenepithelkarzinomen im Kopf-Hals-Bereich. *HNO* **37**: 454–459

Kronberger L Jr, Weblacher M, Estelberger W, Hauser H, Schauenstein E, Schauenstein K, Smola M and Steindorfer P (1994) Pre- and postoperative analysis of serum IgG1 in patients with benign and malignant breast diseases. In *2nd European Congress on Senology,* Kubista E, Staffen A and Zielinski Ch (eds) pp 479–485. Monduzzi Editore, International Proceedings Division, Bologna, Italy

Maninger K, Schauenstein K, Weblacher M, Tillian H, Schauenstein E (1994) Shift in IgG subclasses – a sensitive early marker for malignant proliferation in tumor bearing rats as a representative model for human cancer. *Cancer Mol Biol* (CMB) **1**: 165–171

Maninger K, Weblacher M, Zatloukal K, Estelberger W, Schauenstein K and Schauenstein E (1994) IgG1 – as the only subclass of human serum IgG – spontaneously undergoes $O_2^-$-induced, noncovalent self-aggregation upon storage at room temperature. *Free Radical Biol Med* **20**: 263–270

Maran AGD, Wilson JA and Gaze MN (1993) The nature of head and neck cancer. *Europ Arch Oto-Rhino-Laryngol* **250**: 127–132

Schauenstein E, Dachs F, Reiter M, Gombotz H and List W (1986) Labile disulfide bonds and free thiol groups in human IgG. I. Assignment to IgG1 and IgG2. *Int Arch Allergy Immun* **80**: 174–179

Schauenstein E, Lahousen M, Weblacher M, Steinschifter W, Estelberger W and Schauenstein K (1996) Selective decrease in serum immunoglobulin G1 – a tissue non specific tumour marker detecting early stages of gynecological malignant diseases with high efficiency. *Cancer* **78**: 511–516

Schauenstein E, Rabl H, Steinschifter W, Hirschmann C, Estelberger W and Schauenstein K (1997) Selective decrease of serum immunoglobulin G1 as a marker of malignant transformation in colorectal tissue. *Cancer* **79**: 1482–1486

Silverman NA, Alexander JC and Chretien PB (1976) CEA levels in head and neck cancer. *Cancer* **37**: 2204–2211

UICC TNM (1992) *Classification of malignant tumors*, 4th ed, Sobin LH, Wittekind CH (eds). John Wiley & Sons Inc.: New York

Walther EK, Dahlmann N and Gorgulla HT (1990) Tumormarker bei Patienten mit Kopf-Hals-Karzinomen. *Laryngo-Rhino-Otol* **69**: 271–274

Weblacher M, Leitsberger A, Tillian H, Maninger K, Estelberger W, Schauenstein K and Schauenstein E (1993) A decrease in reactive disulfide bonds of serum IgG signals a characteristic change in the IgG subclass pattern of rats bearing experimental tumors. *Int Arch Allergy Immunol* **102**: 340–346

© Cancer Research Campaign 1999

## References

[PDF_00375] Clasen B., Roettger D., Senekowitsch R., Menz E. (1988). Squamous cell carcinoma antigen (SCC). Derzeitige klinische Wertigkeit eines neuen Tumormarkers bei Kopf-Hals-Karzinomen, Zwischenergebnisse einer prospektiven Studie nach 12 Monaten.. Laryngol Rhinol Otol (Stuttg).

[PDF_00379] Dreyfuss A. I., Clark J. R., Andersen J. W. (1992). Lipid-associated sialic acid, squamous cell carcinoma antigen, carcinoembryonic antigen, and lactic dehydrogenase levels as tumor markers in squamous cell carcinoma of the head and neck.. Cancer.

[PDF_00383] Eibling D. E., Johnson J. T., Wagner R. L., Su S. (1989). SCC-RIA in the diagnosis of squamous cell carcinoma of the head and neck.. Laryngoscope.

[PDF_00386] Fischbach W., Meyer T., Barthel K. (1990). Squamous cell carcinoma antigen in the diagnosis and treatment follow-up of oral and facial squamous cell carcinoma.. Cancer.

[PDF_00389] Gleich L. L., Srivastava L., Gluckman J. L. (1996). Plasma platelet-derived growth factor: preliminary study of a potential marker in head and neck cancer.. Ann Otol Rhinol Laryngol.

[PDF_00392] Kawano Y., Noma T., Yata J. (1994). Regulation of human IgG subclass production by cytokines. IFN-gamma and IL-6 act antagonistically in the induction of human IgG1 but additively in the induction of IgG2.. J Immunol.

[PDF_00395] Koch T., Eiffert H., Spindler M. B. (1989). Die Relevanz des neuen Tumormarkers SCC (Squamous Cell Carcinoma Antigen) für die Diagnostik und Verlaufskontrolle von Plattenepithelkarzinomen im Kopf-Hals-Bereich.. HNO.

[PDF_00409] Maninger K., Weblacher M., Zatloukal K., Estelberger W., Schauenstein K., Schauenstein E. (1996). IgG1--as the only subclass of human serum IgG--spontaneously undergoes O2(-)-induced, noncovalent self-aggregation upon storage at room temperature.. Free Radic Biol Med.

[PDF_00414] Maran A. G., Wilson J. A., Gaze M. N. (1993). The nature of the head and neck cancer.. Eur Arch Otorhinolaryngol.

[PDF_00416] Schauenstein E., Dachs F., Reiter M., Gombotz H., List W. (1986). Labile disulfide bonds and free thiol groups in human IgG. I. Assignment to IgG1 and IgG2 subclasses.. Int Arch Allergy Appl Immunol.

[PDF_00419] Schauenstein E., Lahousen M., Weblacher M., Steinschifter W., Estelberger W., Schauenstein K. (1996). Selective decrease in serum immunoglobulin G1. A tissue nonspecific tumor marker detecting early stages of gynecologic malignant disease with high efficiency.. Cancer.

[PDF_00423] Schauenstein E., Rabl H., Steinschifter W., Hirschmann C., Estelberger W., Schauenstein K. (1997). Selective decrease of serum immunoglobulin G1 as a marker of malignant transformation in colorectal tissue.. Cancer.

[PDF_00426] Silverman N. A., Alexander J. C., Chretien P. B. (1976). CEA levels in head and neck cancer.. Cancer.

[PDF_00430] Walther E. K., Dahlmann N., Gorgulla H. T. (1990). Tumormarker bei Patienten mit Kopf-Hals-Karzinomen.. Laryngorhinootologie.

[PDF_00432] Weblacher M., Leitsberger A., Tillian H., Maninger K., Estelberger W., Schauenstein K., Schauenstein E. (1993). A decrease in reactive disulfide bonds of serum IgG signals a characteristic change in the IgG subclass patterns of rats bearing experimental tumors.. Int Arch Allergy Immunol.

